# Therapy with Cardiomyocytes Derived from Pluripotent Cells in Chronic Chagasic Cardiomyopathy

**DOI:** 10.3390/cells9071629

**Published:** 2020-07-07

**Authors:** Guilherme Visconde Brasil, Danúbia Silva dos Santos, Elias Ataide Mendonça, Fernanda Cristina Paccola Mesquita, Tais Hanae Kasai-Brunswick, Sandro Torrentes da Cunha, Cibele Ferreira Pimentel, Andréia de Vasconcelos-dos-Santos, Rosália Mendez-Otero, Clério Francisco de Azevedo Filho, Regina Coeli dos Santos Goldenberg, Antonio Carlos Campos de Carvalho

**Affiliations:** 1 Carlos Chagas Filho Institute of Biophysics, Federal University of Rio de Janeiro, Rio de Janeiro-RJ 21941-902, Brazil; guilhermevb@gmail.com (G.V.B.); sds.danubia@gmail.com (D.S.d.S.); ataide.mendonca@gmail.com (E.A.M.); fernanda.fermesquita@gmail.com (F.C.P.M.); kasaitais@biof.ufrj.br (T.H.K.-B.); santorrentes@gmail.com (S.T.d.C. ); cibelepimentel@biof.ufrj.br (C.F.P.); dreiavasc@gmail.com (A.d.V.-d.-S.); rmotero@biof.ufrj.br (R.M.-O.); rcoeli@biof.ufrj.br (R.C.d.S.G.); 2 National Center for Structural Biology and Bioimaging - CENABIO, Federal University of Rio de Janeiro, Rio de Janeiro-RJ 21941-902, Brazil; 3 National Institute of Science and Technology for Regenerative Medicine-REGENERA, Federal University of Rio de Janeiro, Rio de Janeiro-RJ 21941-902, Brazil; 4 D’Or Institute for Research and Education, Rio de Janeiro, Rio de Janeiro-RJ 22281-100, Brazil; clerio.azevedo@gmail.com

**Keywords:** Chagas disease, cell therapy, embryonic stem cell, cardiomyocytes, chronic Chagasic cardiomyopathy

## Abstract

Chagas disease discovered more than a century ago remains an incurable disease. The objective of this work was to investigate the therapeutic potential of cardiomyocytes derived from mouse embryonic stem cells (CM-mESC) in a model of chronic Chagasic cardiomyopathy (CCC). Mouse embryonic stem cells (mESC) were characterized, transduced with luciferase, and submitted to cardiac differentiation. CM-mESC were labeled with superparamagnetic iron oxide particles. To induce CCC, mice were infected with Brazil strain trypomastigotes. At 150 days post-infection (dpi), infected animals were treated with CM-mESC or PBS. Cells were detected by magnetic resonance imaging (MRI) and bioluminescence. Cardiac function was evaluated by MRI and electrocardiogram at 150 and 196 dpi. CCC mice showed significant differences in MRI and ECG parameters compared to non-infected mice. However, no differences were observed in contractile and electrical parameters between cell and PBS injected groups, 45 days after cell transplantation. Cells were detected 24 h after transplantation by MRI. CM-mESC bioluminescence tracking demonstrated over 90% decrease in signal 8 days after treatment. Nevertheless, the Infected + CM-mESC group showed a significant reduction in the percentage of collagen fibers when compared to the Infected + PBS group. In conclusion, CM-mESC therapy was not effective in reversing cardiac functional changes induced by Chagas disease despite some improvement in myocardial fibrosis.

## 1. Introduction

Chronic Chagasic cardiomyopathy (CCC) is caused by a protozoan parasite, *Trypanosoma cruzi.* In 1909 Carlos Chagas described the disease, identified the parasite and the transmission mode [1]. Since then thousands of papers have been published [2,3,4], but the physiopathology of the disease is still disputed. The disease has an acute and a chronic phase, sometimes separated by decades. The acute phase is usually asymptomatic or oligosymptomatic, and the chronic phase can include and indeterminate period, where the patient is also asymptomatic or oligosymptomatic. Most infected patients remain in this indeterminate period, but 10–30% evolve to develop gastro-intestinal and/or cardiac symptoms. In Brazil, the cardiac form of the chronic disease is more common and is characterized by a dilated cardiomyopathy. Patients with CCC can develop fatal arrhythmias or progress to congestive heart failure (CHF), where the only possible therapy is heart transplantation [5]. Due to the shortage of donors and complications related to immune suppression, alternative therapies are needed, as for other types of cardiomyopathies that evolve to CHF.

We have previously used bone marrow-derived cells in preclinic and clinic studies in CCC. Although the animal studies were promising [6,7,8,9,10] and the clinical safety trial showed signs of improved cardiac function [11] the efficacy trial did not show additional benefits to conventional therapy for heart failure patients [12]. In 2017, an extensive revision showed that the use of adult stem cells for therapy in heart diseases, seen as a possible solution to the problem, has not achieved satisfactory results so far [13]. Then, several research groups have begun to investigate the use of pluripotent cells.

Pluripotent cells, whether embryonic (ESC) or induced to pluripotency (iPS), have the ability to differentiate into any cell in the body, including cardiomyocytes [14], making it possible to replace cardiomyocytes destroyed by heart disease, something unattainable with the use of adult stem cells.

The present work reports the use of cardiomyocytes derived from embryonic stem cells in a mouse model of CCC.

## 2. Materials and Methods

### 2.1. Cell Culture and Characterization

The mouse embryonic stem cell line (mESCs) E14TG2A generated at the University of Edinburg by Hooper et al. [15] was kindly donated by Dr. Henrique Marques Souza (University of Campinas, Campinas, SP, Brazil). Cells were cultured as previously described [16] and passaged every 3 days by enzymatic dissociation with 0.25% trypsin-EDTA (Gibco). The culture medium was changed daily. For the detection of aneuploidy, chromosome preparation was performed as previously described [16] and 20 metaphases were karyotyped for each sample (*n* = 3). Total RNA was extracted from the cells using the RNeasy Mini Kit (Qiagen, Germantown, MD, USA) following the manufacturer’s instructions. One µg of total RNA was reversely transcribed into cDNA using random primers and High-Capacity Reverse Transcription Kit (Applied Biosystems, Foster City, CA, USA) following the manufacturer’s instructions as previously described [16]. The sequences of primers and sizes of expected products are presented in Supplementary Table S1. The PCR products were analyzed on a 2% agarose gel (Sigma-Aldrich, St Louis, MO, USA) and revealed using ethidium bromide (Sigma-Aldrich).

For immunofluorescence mESCs were fixed in 4% (*v*/*v*) paraformaldehyde for 20 min at room temperature and permeabilized with 0.3% (*v*/*v*) Triton X-100 as previously described [16]. Primary antibodies for octamer-binding transcription factor 3/4 (OCT3/4; Abcam; ab-19857, diluted 1:500) and stage-specific embryonic antigen-1 [SSEA-1 (480); Santa Cruz Biotechnology; sc-21702; diluted 1:100] were used. Secondary antibodies were Cy3-AffinePure Donkey Anti-rabbit (Jackson Immunoresearch, West Grove, PA, USA; 711-165-152, diluted 1:800) and Alexa Fluor^®^ 488 Goat Anti-mouse (Life Technologies, Carlsbad, CA, USA; A21042, diluted 1:400). Nuclei were stained with TO-PRO^®^-3 (Life Technologies; diluted 1:1000), and coverslips were mounted in an antifading solution VECTASHIELDR H-1000 (Vector Laboratories, Burlingame, CA, USA). Fluorescence was observed on a confocal microscope (Zeiss LSM 510 Meta, Berlin, Germany). The specificity of the immunofluorescent staining was assessed for each experimental condition by performing the reaction in the absence of primary antibodies.

### 2.2. mESCs Transduction with Luciferase 2

Lentiviral vector pMSCV.Luc2.T2A.Puro was constructed as previously described [17]. Lentiviral particles containing Luciferase 2 (Luc2) and puromycin resistance genes were produced in HEK 293FT cells with the pMSCV.Luc2.T2A.Puro vector and the accessory vectors *p*∆8.9 and pHDM-VSV-G, using the transfection reagent FuGene 6 (Roche), as previously described [16]. At 24, 36, and 48 h after transfection, culture media containing lentiviral particles were filtered (0.22 µm; Merck Millipore, Darmstadt, Germany) and centrifuged at 20,000× *g*, mESC were cultivated with polybrene (8 µg/mL; Merck Millipore) and lentiviral particles. After 24 h of incubation, culture medium was replaced. Approximately 60 h after transduction, 1.0 µ/mL puromycin was added to the medium. Cells were selected for four days and then expanded for bioluminescence imaging assay.

### 2.3. Cardiac Differentiation of mESC

mESCs were submitted to cardiac differentiation using a protocol adapted from Chen et al. [18]. mESCs were dissociated by 0.25% trypsin-EDTA and cultured using the hanging drop (HD) method to form embryoid bodies (EBs). Approximately 600 cells in each 20 µL drop of differentiation medium [high glucose (4.5 g/L) Dulbecco’s Modified Eagle’s medium (DMEM; Gibco) supplemented with 20% (*v*/*v*) FBS, 50 U/mL penicillin-streptomycin (Gibco), 2 mM L-glutamine (Sigma-Aldrich), 0.1 mM β-mercaptoethanol (Gibco), 1% (*v*/*v*) nonessential amino acids (Gibco), 2 µM dorsomorphin dihydrochloride (Tocris Bioscience) and 1% dimethyl sulfoxide (DMSO; Sigma-Aldrich)] were plated on the lids of 100 mm plates (Corning) and cultured as HD for 2 days. After that, EBs were transferred to 60 mm plates (Corning) coated with poly 2-hydroxyethyl methacrylate (Sigma-Aldrich) and cultured in suspension using a medium with the same specification, except for dorsomorphin. On day 5, EBs were transferred to 0.1% (*v*/*v*) gelatin-coated dishes (35 mm; Corning) and cultured in differentiation medium, without dorsomorphin and DMSO for ten more days. In this step, the culture media was changed every 2 days. On day 15, the cardiomyocytes derived from the mESC (CM-mESC) were obtained.

### 2.4. Flow Cytometry

On the 14th day, differentiated cells were dissociated with 0.25% trypsin-EDTA. For intracellular staining, cells were fixed in 4% paraformaldehyde for 20 min at room temperature and permeabilized with 0.3% Triton X-100 in PBS for 30 min. Cells were blocked with 0.5% (*v*/*v*) BSA in PBS and stained with troponin T cardiac isoform Ab-1 (Thermo Scientific^TM^; diluted 1:200) for 30 min at room temperature. Subsequently, cells were incubated for 30 min at room temperature with Alexa Fluor^®^ 647 Goat Anti-mouse (Life Technologies, Carlsbad, CA, USA; A21236, diluted 1:1000). Permeabilized cells were selected by DAPI staining and samples were analyzed using BD FACSAria IIu (BD Bioscience, San Jose, CA, USA) and FlowJo software version 10.

### 2.5. CM-mESC Labeling with Magnetic Nanoparticles

CM-mESC were labeled with superparamagnetic iron oxide (SPION) particles (FeraTrack Contrast Particles; Miltenyi Biotec, Auburn, CA, USA). The particles were prepared as described by the manufacturer and incubated with the cells in culture for 24 h. The medium was removed and CM-mESC were washed with PBS, dissociated by 0.25% trypsin-EDTA, and transplanted in animals for magnetic resonance imaging (MRI) tracking.

Before transplantation, incorporation of FeraTrack nanoparticles by the CM-mESC was analyzed by immunofluorescence to identify the dextran-coating of the particles, and/or by Prussian blue reaction to detect iron within the cells. For these analyses, the labeled cells were plated on glass coverslips coated with 0.2% (*v*/*v*) gelatin, rinsed in PBS, and fixed in 4% (*v*/*v*) paraformaldehyde for 20 min at room temperature. For immunofluorescence, CM-mESC were permeabilized with 0.3% (*v*/*v*) Triton X-100 as previously described [16]. The primary and second antibodies were anti-dextran (Stem Cell Technologies; 01403, diluted 1:500) and Alexa Fluor^®^ 488 Goat Anti-mouse (Life Technologies; A21042, diluted 1:400), respectively. Nuclei were stained with TO-PRO^®^-3 (Life Technologies, Carlsbad, CA, USA; diluted 1:1000). Fluorescent samples were analyzed under a confocal microscope (Zeiss LSM 510 Meta, Berlin, Germany). For the Prussian blue reaction, cells were washed twice with PBS and incubated with Perl’s’ reagent (20% (*v*/*v*) potassium ferrocyanide and 20% (*v*/*v*) hydrochloric acid in water) for 20 min at room temperature. Cultures were then washed once in PBS, dehydrated through a graded ethanol series, and mounted with Entellan (Merck KGaA, Darmstadt, Germany). The samples were observed by phase-contrast optic microscopy (Olympus, Center Valley, PA, USA).

### 2.6. Animals

8–10 weeks old adult female and male CD1 mice (*n* = 63) were obtained from Carlos Chagas Filho Biophysics Institute (IBCCF, UFRJ, Rio de Janeiro, Brazil). All experiments were performed in conformity with the guidelines of the National Council for the Control of Animal Experimentation (Brazil) and the National Institutes of Health (NIH) guide for the care and use of laboratory animals. This study was approved by the local Ethics Committee on the Use of Animals in Scientific Experimentation (Health Science Centre of the Federal University of Rio de Janeiro), under protocol number 163/13. The animals were housed in our animal facility at the National Center for Structural Biology and Bioimaging (CENABIO-UFRJ, Rio de Janeiro, Brazil) with temperature-controlled (23 °C), 12/12 h light-dark cycle and access to standard mouse chow and water ad libitum.

### 2.7. Infection with T. cruzi and Cell Transplantation

CD1 mice (*n* = 50) were submitted to intraperitoneal inoculation of 3 × 10^4^ bloodstream trypomastigotes from the Brazil strain of *T. cruzi*. To evaluate parasitemia, tail vein blood was collected from 5 random infected animals every 3 days from the 5th to the 44th day post-infection. Blood samples (10 µL) were diluted in ammonium chloride 0.85% and parasites were counted using a Neubauer chamber and optical microscopy (Olympus). Non-infected controls mice (*n* = 13) were submitted to the same blood collection procedure. To evaluate survival rate, the animals were observed twice a day. Since the exact time-point that separates the end of the acute phase and the beginning of the chronic Chagas disease is not well defined, we considered mice that survived as chronic Chagasic animals.

**Figure 1:**
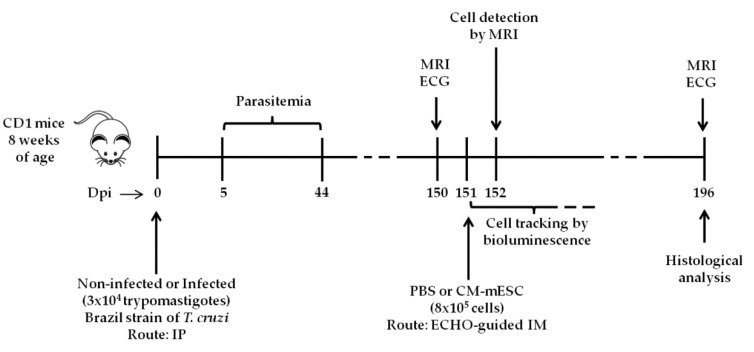
Schematic illustration of the experimental design. CM-mESC—cardiomyocytes derived from mouse embryonic stem cells, ECG—electrocardiogram, ECHO—echocardiogram, Dpi—days post-infection, IM—intramyocardial, IP—intraperitoneal, MRI—magnetic resonance imaging.

At 150 days post-infection, out of 50 infected animals, 36 died. The 14 remaining mice were distributed into two groups (Infected + PBS group; *n* = 7 and Infected + CM-mESC group; *n* = 7) and treated using echocardiogram-guided intramyocardial injections with 30 µL of PBS containing either 8 × 10^5^ CM-mESC or just PBS. The non-infected controls mice (Non-infected group; *n* = 13) were not submitted to this procedure. The study design is shown in Figure 1.

The echocardiogram-guided intramyocardial injections were performed using a 30 MHz ultrasound scan head connected to the Vevo 770 Imaging System (*VisualSonics*). For this, mice were anesthetized with 1.5% isoflurane and heart rate and body temperature were monitored during the procedure.

### 2.8. Assessment of Cardiac Performance

MRI was performed on a 7.0 T, 210 Bore Actively Screened Refrigerated Magnet System (*Varian, Inc. NMR Systems)* under inhalation anesthesia (1.5% isoflurane in O_2_), body temperature maintained at 36.5 °C and heart rate between 450–500 beats per minute (bpm). High-resolution bright-blood MRI experiments were conducted using an ECG-triggered fast low-angle shot (FLASH) gradient-echo (GE) pulse sequence tailored for murine imaging. Hearts were imaged from the base to the apex by a stack of two-dimensional images. The electrocardiographic gating was optimized with two subcutaneous precordial leads with respiratory motion and body temperature monitors (SA Instruments). The scanning parameters were optimized for the signal-to-noise ratio (SNR) as follows: flip angle = 15°, echo time (TE) = 1.9 ms, repetition time (TR) ≅ R-R interval, radiofrequency (RF) pulse width = 1.0 ms, number of averages = 10 and 15 frames per heart cycle were obtained. All images were acquired with a field of view (FOV) of 30 × 30 mm and a data matrix of 128 × 128 mm. The total scan time was in the range of 60 min per animal. Each imaging protocol resulted in five to seven 1 mm thick short-axis images covering the whole heart from apex to base with no gap between slices. Data were analyzed with OsiriX DICOM viewer software (Pixmeo). Ventricular slice volumes were determined from end-diastolic and end-systolic images by multiplication of compartment area and slice thickness. End-diastolic volume (EDV) and end-systolic volume (ESV) were calculated as the sum of all slices and ejection fraction (EF) was calculated by Simpson’s rule for both the left and right ventricles.

Electrocardiogram (ECG) signals were recorded using Power Lab/400 (*AD Instruments,* São Paulo, SP, Brazil) and electrical activity parameters (P-wave duration, PR interval, QRS duration, and corrected QT interval) were analyzed using Lab Chart 7.3 software (*AD Instruments*). For ECG examinations, mice were gently placed on the ECG recording platform equipped with three electrodes configured to contact the underside of their paws. ECG signals were collected for 5 min per mouse and approximately 1500 consecutive heartbeats were analyzed.

### 2.9. Cell tracking by MRI and Bioluminescence

To confirm the presence of CM-mESC inside the left ventricular wall, cells labeled with SPION particles were tracked in vivo by MRI measurements at 24 h. Images were acquired before and after the echocardiogram-guided intramyocardial injections. Data were processed with the use of OsiriX DICOM viewer software.

Bioluminescence imaging assay was performed as described [19]. For in vitro studies, transduced cells were plated in a 24-well plate at different concentrations: 4 × 10^4^, 6 × 10^4^, 8 × 10^4^, 10 × 10^4^, and 12 × 10^4^ CM-mESC per well. D-Luciferin (150 μg/mL) (Promega Corporation) was added to the culture medium following the guidelines of the manufacturer. The culture plate was immediately positioned in the IVIS Lumina Imaging System (Caliper Life Sciences) and images were acquired after a 10 s exposure period. For in vivo studies, mice received D-luciferin (150 mg/kg) intraperitoneally. Ten minutes after injection, they were anesthetized with 1.5% of isoflurane in O_2_ and placed in the IVIS Lumina Imaging System. Image acquisitions were performed 1, 2, 4, 6, and 8 days after the injection of transduced CM-mESCs. The exposure time was 3 min.

### 2.10. Histology

On day 196 (corresponding to 45 days after PBS or cell transplant), all mice were euthanized, hearts were washed in PBS, and fixed in paraformaldehyde 4% for 24 h. Cardiac tissue was embedded in paraffin and 8 μm slices were obtained. Slices were stained with hematoxylin-eosin (HE) or Sirius red. The quantification of the inflammatory or fibrous process was performed by analyzing 10 or 30 random fields of magnification (40×), respectively, covering both ventricles, from the apical to basal regions. The number of inflammatory cells were determined using Image J software and the fibrotic area were determined using Image-Pro Plus 5.0 software. The mean number of inflammatory cells or the fibrosis percentage of the 10 or 30 fields was used for statistical comparison between experimental groups.

### 2.11. Statistical Analysis

All data are presented as mean ± standard derivation. Cardiac performance parameters and histology were analyzed using the Two-way analysis of variance (ANOVA) followed by Tukey’s comparison test. The survival analysis was evaluated using Kaplan–Meier curves and analyzed with the Gehan Breslow–Wilcoxon test. All analyses were performed with GraphPad Prism software, version 6.1 (GraphPad Software, Inc., LA Jolla, CA, USA), and *p* < 0.05 was considered significant.

## 3. Results

### 3.1. mESC Culture and Differentiation

mESC colonies exhibited typical morphology of undifferentiated embryonic stem cells, including bright edges, high ratio of nucleus to the cytoplasm, prominent nucleoli, and close spacing between the cells (Figure S1A). Karyotype analysis showed a normal karyotype, with 40 chromosomes (Figure S1B). In order to confirm the pluripotency of these cells, the amplification of certain marker genes (*Oct3/4*, *Sox-2,* and *Nanog*) was detected by RT-PCR (Figure S1C) and the expression of Oct3/4 and SSEA-1 was observed by immunofluorescence (Figure S1D).

Next, mESC were submitted to cardiac differentiation protocol. After 2 days, the EBs were generated by HD culture (Figure S1E). After 7 days, we observed EBs adhered to and cells migrating from the central region to the periphery (Figure S1F). In addition, some differentiated cells exhibited spontaneous contraction. On the 14th day, the efficiency of the cardiac differential protocol was evaluated by flow cytometry. We detected high percentages of cardiac troponin T-positive cells (Figure S1G).

### 3.2. Analysis of Parasitemia and Survival

**Figure 2:**
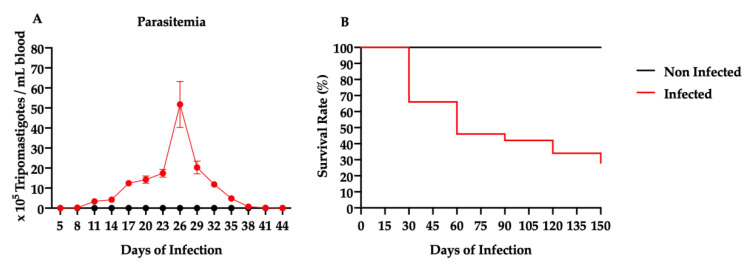
Parasitemia and survival of CD1 mice (**A**) The number of parasites (×10^5^) per mL of blood versus days after infection progress. (**B**) Kaplan–Meier curve showing survival of infected mice with *T. cruzi* (Brazil strain) and non-infected animals until 150 days post-infection (dpi). Mice in the infected group showed a survival rate of 28%. None of the non-infected mice died.

The parasitemia was followed from the 5th to the 44th day post-infection. As shown in Figure 2A, the peak of parasitemia occurred around the 26th day and no parasite was detected after 44 days post-infection. In Figure 2B, survival analysis showed that the highest percentage of death (34%) in infected mice occurred during the acute phase of Chagas disease. Sixty days post-infection, the survival rate was 46%. On the 150th day post-infection, only 28% of the mice survived and the 14 animals were randomly allocated to the placebo (PBS treated) and cell groups. All animals from the non-infected group survived.

### 3.3. Cardiac Functional Evaluation Post CM-mESC Treatment

**Figure 3:**
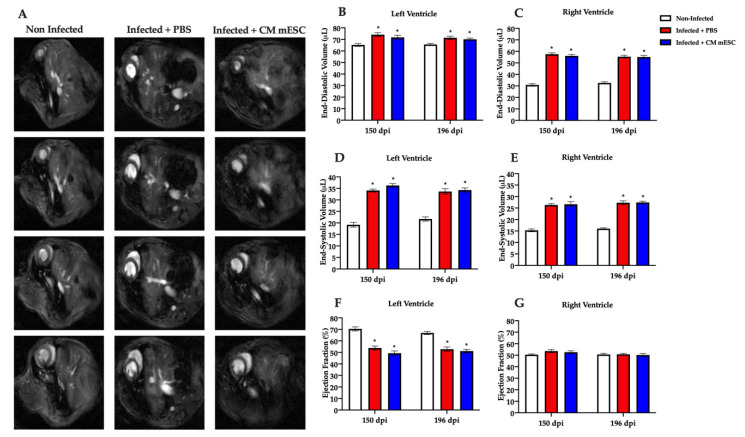
Cardiac function evaluation by magnetic resonance imaging (MRI). Measures were performed 150 and 196 (corresponding to 45 days after the injection of CM-mESC or PBS in infected mice) days post-infection (dpi). (**A**) Representative images in diastole. A sequence of images from the same animal on day 196 is shown in each column. (**B**–**G**) Analysis of left and right ventricle end-diastolic volume (EDV) (**B**,**C**), end-systolic volume (ESV) (**D**,**E**), and ejection fraction (EF) (**F**,**G**), respectively. Two away ANOVA with Tukey’s comparison test: * *p* < 0.05 compared to the Non-infected group. Data shown as mean ± standard error of mean; *n* = 13 for Non-infected group, *n* = 7 for Infected + PBS group, and *n* = 7 for Infected + CM-mESC group. CM-mESC—cardiomyocytes derived from mouse embryonic stem cells.

To evaluate the potential benefits of CM-mESC therapy, we performed analyses of cardiac mechanical and electrical functions. Figure 3A shows representative mice MRI from all three groups: non-infected, infected treated with PBS, and infected treated with CM-mESC. On day 150, we observed significant EDV increase in both left and right ventricles of the Infected + PBS and Infected + CM-mESC animals when compared to the Non-infected group (Figure 3B,C), indicating cavities dilatation, which was more pronounced in the right ventricle. In addition, left and right ventricular ESV were also increased in infected animals (Figure 3D,E), suggesting that systolic function was compromised. Indeed, the left ventricular EF was reduced in the infected mice when compared to non-infected animals (Figure 3F). However, no differences were observed in the right ventricular EF among groups (Figure 3G).

On day 196 of the study (corresponding to 45 days after PBS or cell therapy), no differences were observed in the EDV, ESV, and EF of the ventricles between Infected + PBS and Infected + CM-mESC mice. Besides, we observed that differences remained significant in these parameters when compared with the Non-infected group (Figure 3B–G).

**Figure 4:**
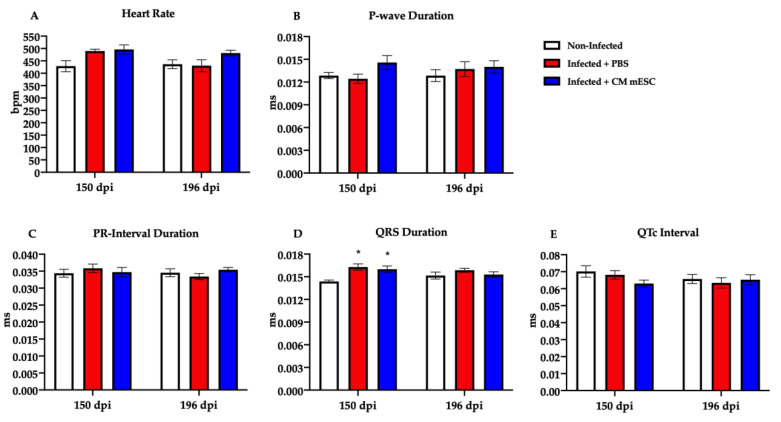
Electrical activity evaluation by electrocardiogram (ECG). Measures were performed 150 and 196 (corresponding to 45 days after the injection of CM-mESC or PBS in infected mice) days post-infection (dpi). (**A**–**E**) Analysis of heart rate (**A**), PR interval (**B**), P-wave duration (**C**), QRS duration (**D**), and QTc interval (**E**). Two away ANOVA with Tukey’s comparison test: * *p* < 0.05 compared to the Non-infected group. Data shown as mean ± standard error of mean; *n* = 13 for Non-infected group, *n* = 7 for Infected + PBS group, and *n* = 7 for Infected + CM-mESC group. CM-mESC—cardiomyocytes derived from mouse embryonic stem cells, QTc—corrected QT interval.

The ECG analyses at 150 days revealed that the infected groups had a significant increase in QRS duration when compared to non-infected mice (Figure 4D). However, no differences were observed in this parameter 45 days after cell transplantation. Moreover, we did not observe significant alterations in Heart Rate, P-wave duration, PR interval, and corrected QT interval among groups at both times-point of this study (Figure 4A–C,E).

### 3.4. Distribution of CM-mESC after Cell Therapy

#### 3.4.1. Cell Detection by MRI

**Figure 5:**
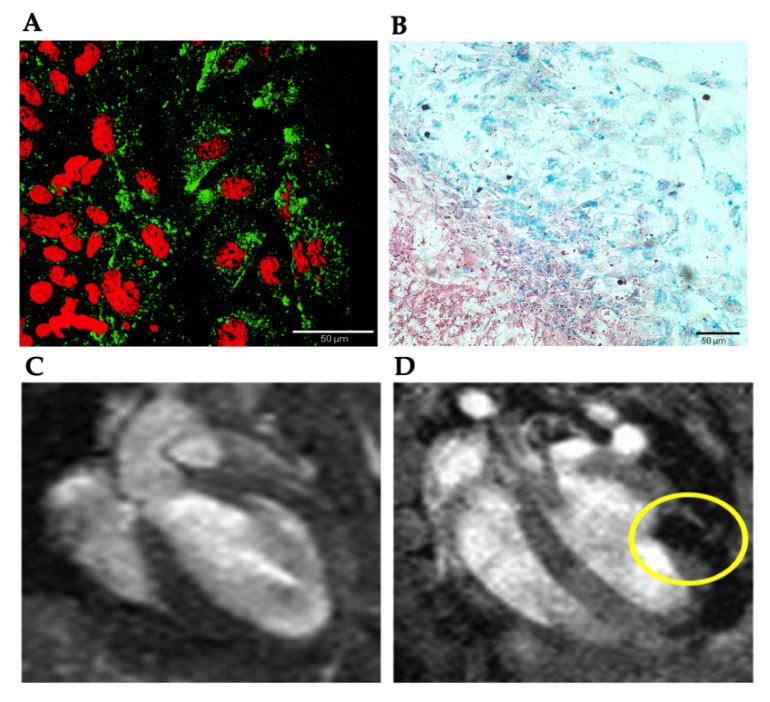
Detection of FeraTrack-labeled CM-mESC. (**A**) Micrograph showing anti-dextran (green) staining in labeled CM-mESC. The nuclei were labeled with Topro (red). (**B**) Iron from SPION particles is visible after the Prussian blue reaction. Representative images of in vivo MRI before (**C**) and 24 h after (**D**) cell transplantation. Yellow circle indicates the hypointense (black) spots corresponding to FeraTrack-labeled CM-mESC in the free left ventricular wall. No signal was detected before cell therapy. CM-mESC - cardiomyocytes derived from mouse embryonic stem cells, SPION - superparamagnetic iron oxide. Scale bar: 50 µm.

CM-mESC were labeled with SPION and the incorporation of the FeraTrack nanoparticles by these cells was evaluated in vitro. Figure 5A,B shows the presence of SPION in the CM-mESC. Since the particles are coated with dextran, it is possible to reveal them with a specific antibody (Figure 5A). Also, the iron in the SPION can be detected in FeraTrack-labeled CM-mESC by the Prussian blue reaction (Figure 5B).

To confirm that CM-mESC were injected into the myocardium and remained in the heart, in vivo analysis by MRI was performed. Figure 5 shows MRI images before and 24 h after echocardiogram-guided intramyocardial injections of FeraTrack-labeled CM-mESC. The signal of the labeled cells was visible as a dark stain in the free left ventricular wall 24 h after cell transplantation, demonstrating the exact injection site (Figure 5D). No signal was detected in the heart before CM-mESC therapy (Figure 5C).

#### 3.4.2. Cell Detection by Bioluminescence Assay

**Figure 6:**
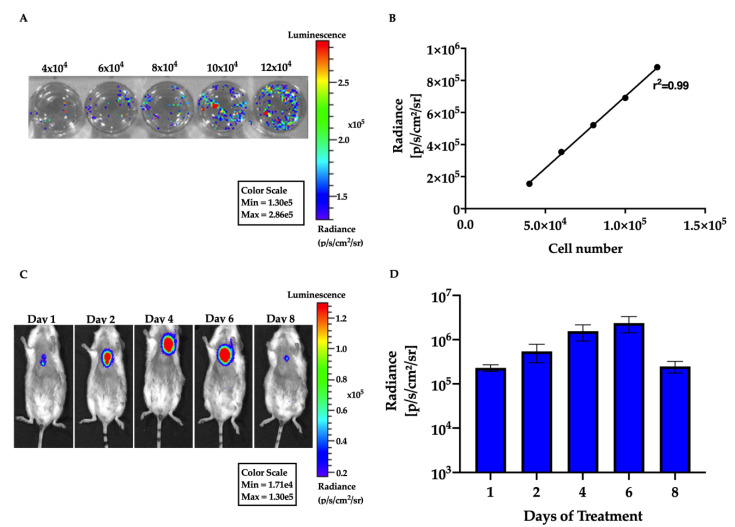
CM-mESC tracking by bioluminescence. (**A**) Assessment of luminescent signal intensity in CM-mESC transduced with the luciferase 2 gene in vitro. The numbers shown above the wells indicate the number of cells. (**B**) The signal linearly increased with cell numbers (*r*^2^ = 0.99). (**C**) in vivo cell tracking shows a luminescent signal on the precordial region in Chagasic mice (*n* = 7). Luminescence was detected for up to 8 days after injection. (**D**) Quantification of the luminescent signal showed a progressive decrease in radiance units with time. The data is plotted on a log scale. CM-mESC—cardiomyocytes derived from mouse embryonic stem cells.

CM-mESC were transduced with the Luciferase 2 gene and the luminescent signal was evaluated in vitro. Imaging of cells containing a range of 4 × 10^4^–12 × 10^4^ transduced cardiomyocytes showed a strong linear correlation between cell number and the luminescent signal (r^2^ = 0.99; Figure 6A,B), suggesting that this approach could be reliably used to track and quantify cell distribution in vivo. The bioluminescent signal of CM-mESC-Luc2 was detected in the thoracic region of animals in the Infected + CM-mESC group until day 8 post-injection (Figure 6C). Radiance quantification demonstrated a 90% decrease in the mean bioluminescent signal at 8 days after cell injection (Figure 6D).

Recently, our group demonstrated that CM-mESC transplanted in immunosuppressed CD1 mice were also only detected until the 8th-day post-injection [16]. This strongly suggests that immune rejection was not the cause of engraftment failure in the present study.

### 3.5. Quantitative Assessment of Myocardial Collagen

**Figure 7:**
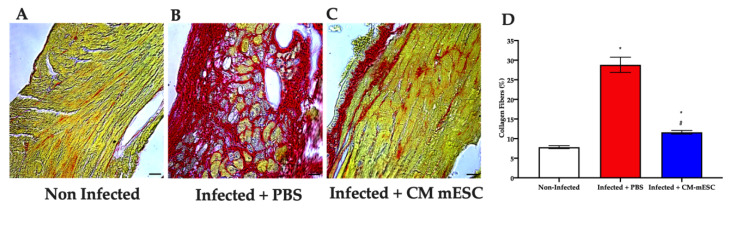
Histological analyses of fibrosis. (**A**–**C**) Representative images of histological cardiac sections stained with Sirius red: (**A**) Non-infected group, (**B**) Infected + PBS group, and (**C**) Infected + CM-mESC group 45 days after cell transplantation. (**D**) The graph shows that the Infected + PBS group exhibited a higher percentage of collagen fibers when compared to the Non-infected group. CM-mESC therapy attenuated significantly the percentage of collagen compared to the PBS treated group. One away ANOVA with Tukey’s comparison test: * *p* < 0.05 compared to the Non-infected group; # *p* < 0.05 compared to the Infected + PBS group. Data are shown as mean ± standard error of the mean; *n* = 5 for each group. CM-mESC—cardiomyocytes derived from mouse embryonic stem cells. Scale bar: 20 µm.

The potential benefits of CM-mESC therapy were also evaluated by histological analyses of Sirius red-stained cardiac sections. The results of these analyses demonstrated that the Infected +PBS mice had a higher percentage of collagen fibers in the myocardium than Non-infected group. However, this increase was significantly attenuated after the CM-mESC transplantation (Figure 7).

### 3.6. Quantitative Assessment of Myocardial Inflammation

**Figure 8:**
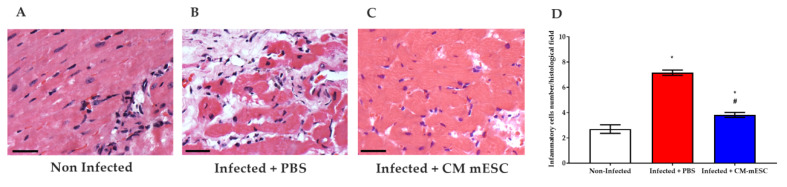
Analysis of inflammatory cells. (**A**–**C**) Representative images of histological cardiac sections stained with hematoxilin-eosin: (**A**) Non-infected group, (**B**) Infected + PBS group, and (**C**) Infected + CM-mESC group 45 days after cell transplantation. (**D**) The graph shows that the Infected + PBS group exhibited a higher number of inflammatory cells when compared to the Non-infected group. CM-mESC therapy attenuated significantly the number of inflammatory cells compared to the PBS treated group. One away ANOVA with Tukey’s comparison test: * *p* < 0.05 compared to the Non-infected group; # *p* < 0.05 compared to the Infected + PBS group. Data are shown as mean ± standard error of the mean; *n* = 5 for each group. CM-mESC—cardiomyocytes derived from mouse embryonic stem cells. Scale bar: 50 µm.

Inflammation was evaluated by histological analyses of HE cardiac sections. The results demonstrated that the Infected + PBS mice had higher numbers of inflammatory cells in the myocardium than the Non-infected group. However, this increase was significantly attenuated after the CM-mESC transplantation (Figure 8).

## 4. Discussion

Cell therapies using adult stem cells have failed to significantly improve cardiac function in clinical trials, despite promising results in small and large animal models [20]. Given that pluripotent stem cells can differentiate into any cell type of our body, the use of ESC or iPS has attracted much attention after adult stem cells failed to show efficacy in human heart diseases. However, the safety hurdles are much greater when using these cells in humans, due to their high proliferative and differential potential [21]. Nevertheless, many groups have attempted to use differentiated cells derived from pluripotent stem cells to treat different diseases in various animal models [22].

In CCC, we have tested bone marrow-derived cells in rodents with promising results [6,7,8,9,10], but clinical trials failed to show any additional benefit to standard therapy [12]. Therefore, we decided to test CM-mESC in a mouse model of CCC. The rationale was that these cardiomyocytes could replace the ones lost to the parasite-mediated disease, in much the same way as reported in models of ischemic heart disease [23,24,25]. Non-human primates and pigs subject to myocardial infarction have been treated with cardiomyocytes derived either from human ESC or iPS, under immunosuppressive regimen [23,24,25]. The results showed robust engraftment of human cells in the hearts up to three months after cell injection and, significant improvement of cardiac function. In the current study, we did not see functional improvement in heart function after cell therapy and this could have resulted from the allogeneic nature of the mouse embryonic stem cell line, derived from 129/Ola mice, while our murine CCC model used CD1 mice. Furthermore, due to parasite persistence, we could not use immunosuppression to avoid CM-mESC rejection. Thus, the lack of functional effects could be attributed to immune cell rejection, compatible with the short survival of the injected cells as evidenced by the bioluminescence imaging. However, in a previous publication, we used these same CM-mESC cells in non-infected CD1 mice immunosuppressed with cyclosporine and could also not detect a bioluminescent signal above background 8 days after cell injection [16], as shown in Figure 6. The limited survival of transplanted cells into the murine heart has been described by many authors irrespective of the use of syngeneic or allogeneic cells, of immunosuppressants and, of cell type [17,26,27,28,29]. Thus, even though robust engraftment of human pluripotent stem cell-derived cardiomyocytes has been described in non-human primates [24,25] subject to myocardial infarction, we could not detect engraftment of CM-mESC in the murine CCC model. These discordant results may be the result of interspecies differences since most murine cardiac disease models cited above did not show significant cell engraftment, irrespective of the disease model used.

We previously reported an improvement in heart function using these same cells in a doxorubicin-induced heart failure model in CD1 mice [16]. Since the CM-mESC did not engraft and survived for a little over one week the only way they could have improved heart function was through paracrine secretion of protective factors. In fact, functional recovery from myocardial infarction has been reported using extracellular vesicles secreted by cardiomyocytes derived from iPS cells [30] and ESC-derived cardiovascular progenitor cells [31] in murine hearts. Unfortunately, in the current CCC model, we did not observe improvements in mechanical or electrical properties of the cell treated animals, even though injecting the same number of cells and using the same route as in the doxorubicin model mentioned above. As to the lack of effect of cell therapy in the CCC model compared to the doxorubicin model, one can speculate that the duration of the cardiac insult may be determinant of the therapy success. In the doxorubicin, model cells were injected 26 days after the last doxorubicin injection [16], while in the CCC model the cell therapy was applied 150 days after parasite infection. We acknowledge that injecting the cells at different time points after infection might produce distinct functional outcomes.

Although we could not detect functional improvement, surprisingly, the cell treated infected mice had significantly lower percentages of collagen fibers and inflammatory cells than the PBS treated infected mice. We would expect this difference in fibrosis and inflammation to somehow reflect in the functional properties of the heart, but intriguingly this was not observed. We attribute the decreased fibrosis to the diminished inflammation, since CCC is considered an inflammatory cardiomyopathy [32]. In fact, using a highly sensitive bioluminescence assay, Lewis et al. [33] showed that chronically infected mice develop myocarditis and cardiac fibrosis despite not detecting parasites in the heart.

In summary, the use of CM-mESC did not improve cardiac function in a murine model of CCC. We propose that shorter intervals from prime infection and larger animal studies should be performed before we exclude pluripotent derived cardiomyocytes as a cell of choice for therapy in CCC. The dog is a good model for CCC and deriving canine iPS has already been achieved. Given the differences in cell engraftment between the murine and primate and pig models cited above, we think it is reasonable to test these cells in a larger animal model of Chagas disease before discarding them as a therapeutic option.

## 5. Conclusions

Cell therapy using cardiomyocytes derived from mouse embryonic stem cells was not efficacious in preventing cardiac dysfunction induced by infection with the parasite *Trypanosoma cruzi* in a murine model of chronic Chagasic cardiomyopathy. The injected cells did not engraft, and their paracrine effect was not sufficient to improve cardiac function, although they significantly reduced the percentage of collagen fibers in the heart compared to PBS injection.

## Supplementary Materials

The following are available online at https://www.mdpi.com/2073-4409/9/7/1629/s1, Figure S1: Characterization and differentiation of mESC. Table S1: Primers sequences for mESC pluripotency gene expression.
