# Supplementary material for: Therapy with Cardiomyocytes Derived from Pluripotent Cells in Chronic Chagasic Cardiomyopathy
Source: Cells. 2020 Jul 7;9(7):1629. doi: 10.3390/cells9071629 (PMC7408395; doi:10.3390/cells9071629)
Supplement: Supplementary file 1 [file cells-09-01629-s001.pdf]

Supplementary Material

Therapy with cardiomyocytes derived from pluripotent cells in chronic chagasic cardiomyopathy

Guilherme Visconde Brasil[1†], Danúbia Silva dos Santos[1†], Elias Ataide Mendonça[1], Fernanda Cristina Paccola Mesquita[1], Tais Hanae Kasai Brunswick[1,2,3], Sandro Torrentes da Cunha[1], Cibele Ferreira Pimentel[1], Andreia de Vasconcelos-dos-Santos[1], Rosália Mendez-Otero[1,3], Clério Francisco de Azevedo Filho[4], Fernanda Freire Tovar Moll[3,4], Regina C. S. Goldenberg[1,3] and Antonio C. Campos de Carvalho[1,2,3*]

[1]Carlos Chagas Filho Institute of Biophysics, Federal University of Rio de Janeiro, Rio de Janeiro, Brazil
[2]National Center for Structural Biology and Bioimaging - CENABIO, Federal University of Rio de Janeiro, Rio de Janeiro, Brazil.
[3]National Institute of Science and Technology for Regenerative Medicine-REGENERA, Federal University of Rio de Janeiro, Rio de Janeiro, Brazil.
[4] D'Or Institute for Research and Education, Rio de Janeiro, Rio de Janeiro, Brazil.

* Correspondence: acarlos@biof.ufrj.br;
[†] These authors have contributed equally to this work.

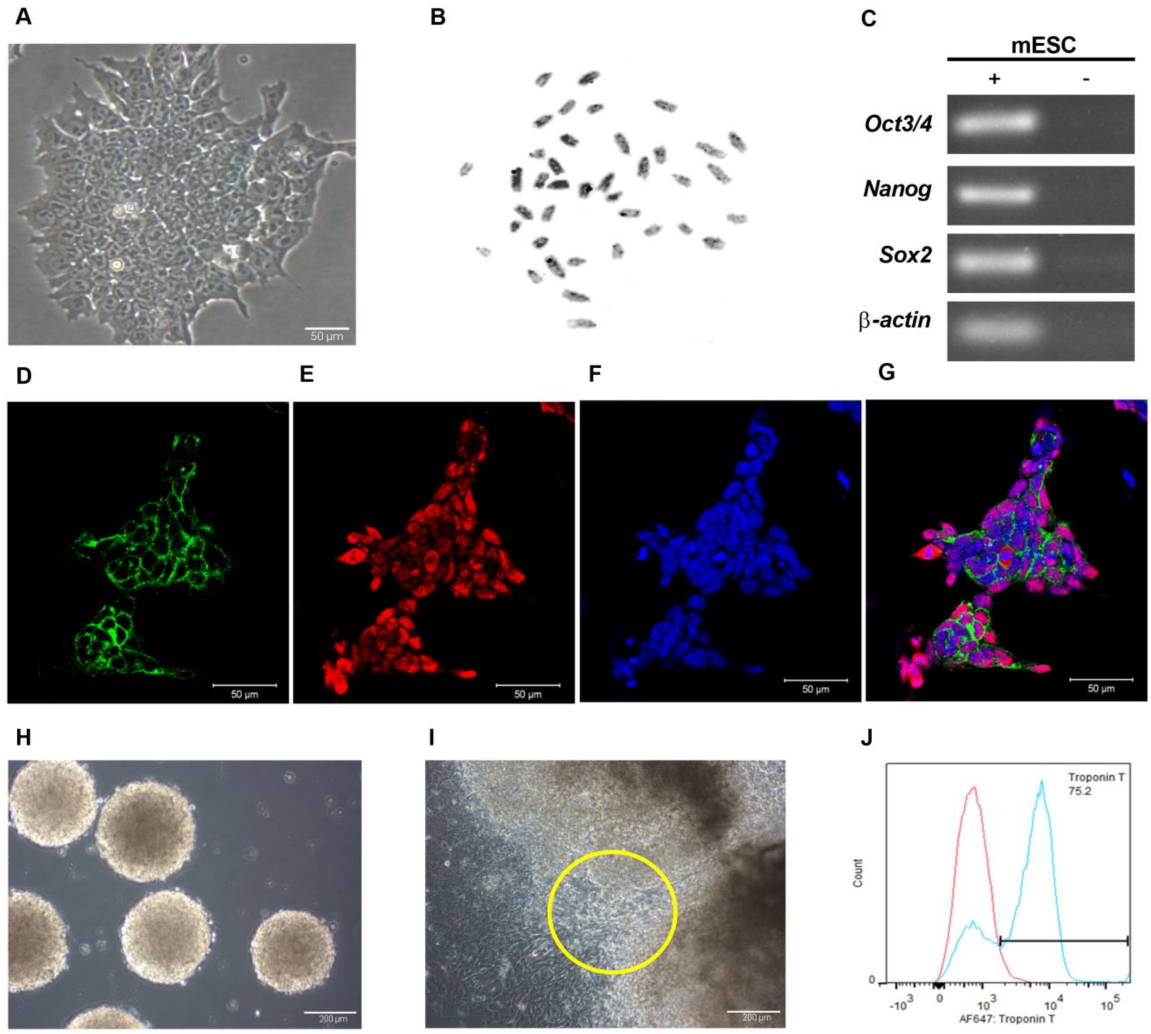

Figure S1. Characterization and differentiation of mESC. (**A**) Undifferentiated mESC line E14TG2A in culture. (**B**) Normal karyotype (40 chromosomes). (**C**) RT-PCR of the transcription factors *Oct3/4*, *Sox2*, and *Nanog* in mESC. Expression of SSEA-1 **(D)** and Oct3/4 **(E)** in mESC by immunofluorescence. The nuclei were labeled with Topro **(F)**. Images overlay **(G)**. (**H**) Embryoid bodies (EBs) formation 2 days after the differentiation. (**I**) Adhered EBs after 7 days of differentiation. Some cells exhibited spontaneous contraction (yellow circle). (**J**) Cardiac troponin T expression was analyzed by flow cytometry in differentiated cells. Histogram overlay differentiated cells stained with secondary antibody (blue) and cells positive for cardiac troponin T (red). mESC mouse embryonic stem cells. Scale bars: (**A, D-G)** 50 µm; (**H, I**) 200 µm.

Table S1. Primers sequences for mESC pluripotency gene expression.

Gene product	Sequence	Size (base pairs)
Oct3/4	F - AGCCTGAGGGCGAAGCAGGA<br>R - CCCCAGGGTGAGCCCCACAT	236
Nanog	F - CAGCCCTGATTCTTCCACCAGTCCC<br>R - TGGAAGGTTCCCAGTCGGGTTCACC	391
Sox2	F - AGCTACAGCATGATGCAGGA<br>R - GGTCATGGAGTTGTACTGCA	126
β-actin	F - CATCACTATTGGCAACGAGCG<br>R - ATGGATGCCACAGGATTCCA	85

F - forward; R - reverse.
